# Development of a Culturally Appropriate Bilingual Electronic App About Hepatitis B for Indigenous Australians: Towards Shared Understandings

**DOI:** 10.2196/resprot.4216

**Published:** 2015-06-10

**Authors:** Jane Davies, Sarah Bukulatjpi, Suresh Sharma, Luci Caldwell, Vanessa Johnston, Joshua Saul Davis

**Affiliations:** ^1^ Menzies School of Health Research Global and Tropical Health Charles Darwin University Darwin Australia; ^2^ Royal Darwin Hospital Infectious Diseases Department Division of Medicine Darwin Australia; ^3^ Miwatj Health Aboriginal Corporation East Arnhem Land Australia; ^4^ Royal Darwin Hospital Chronic Disease Co-ordination Unit Division of Medicine Darwin Australia; ^5^ Dreamedia Darwin Australia; ^6^ Centre for Disease Control Department of Health Darwin Australia

**Keywords:** culture, development, health literacy, hepatitis B, indigenous population, language, portable electronic apps

## Abstract

**Background:**

Hepatitis B is endemic in Indigenous communities in Northern Australia; however, there is a lack of culturally appropriate educational tools. Health care workers and educators in this setting have voiced a desire for visual, interactive tools in local languages. Mobile phones are increasingly used and available in remote Indigenous communities. In this context, we identified the need for a tablet-based health education app about hepatitis B, developed in partnership with an Australian remote Indigenous community.

**Objective:**

To develop a culturally appropriate bilingual app about hepatitis B for Indigenous Australians in Arnhem Land using a participatory action research (PAR) framework.

**Methods:**

This project was a partnership between the Menzies School of Health Research, Miwatj Aboriginal Health Corporation, Royal Darwin Hospital Liver Clinic, and Dreamedia Darwin. We have previously published a qualitative study that identified major knowledge gaps about hepatitis B in this community, and suggested that a tablet-based app would be an appropriate and popular tool to improve this knowledge. The process of developing the app was based on PAR principles, particularly ongoing consultation, evaluation, and discussion with the community throughout each iterative cycle. Stages included development of the storyboard, the translation process (forward translation and backtranslation), prelaunch community review, launch and initial community evaluation, and finally, wider launch and evaluation at a viral hepatitis conference.

**Results:**

We produced an app called “Hep B Story” for use with iPad, iPhone, Android tablets, and mobile phones or personal computers. The app is culturally appropriate, audiovisual, interactive, and users can choose either English or Yolŋu Matha (the most common language in East Arnhem Land) as their preferred language. The initial evaluation demonstrated a statistically significant improvement in Hep B-related knowledge for 2 of 3 questions (*P*=.01 and .02, respectively) and overwhelmingly positive opinion regarding acceptability and ease of use (median rating of 5, on a 5-point Likert-type scale when users were asked if they would recommend the app to others).

**Conclusions:**

We describe the process of development of a bilingual hepatitis B-specific app for Indigenous Australians, using a PAR framework. The approach was found to be successful with positive evaluations.

## Introduction

### Overview

Chronic hepatitis B (CHB) is endemic in the Indigenous communities of the Northern Territory (NT) of Australia with prevalence rates estimated to be between 3% and 14.2% [1-7], compared with 1% in Australia as a whole [8]. Despite the availability of effective, government subsidized treatments, only 25% of all individuals living with CHB in Australia are estimated to be receiving guideline-based care, with only 5% receiving antiviral therapy [9]. This disparity in rates of hepatitis B and low uptake of treatment is also seen in other Indigenous populations across the world [10,11].

The barriers to Indigenous Australians accessing care for CHB are multifactorial but mainly include the following: gaps in knowledge, low health literacy, ineffective cross-cultural communication, and logistical challenges in accessing the appropriate care. The challenges of effective cross-cultural communication in the context of remote Indigenous communities of the NT have been documented extensively [12-15] with miscommunication said to be pervasive and language translation felt to be only part of the problem. Although many health promotion or information resources exist for hepatitis B [16], the Australian National Hepatitis B strategy [17] and a number of other studies [18-22] specifically highlight the lack of culturally appropriate resources available to facilitate shared understandings of hepatitis B for Indigenous Australians.

We have previously described the results of a qualitative study exploring the knowledge, perceptions, and experiences of remote dwelling Indigenous adults and their health care providers relating to hepatitis B infection [23]. User preferences from this study included the preference for an electronic format with a predominance of pictures; sufficient medical details; human-like figures, not animal analogies; to be in *Yolŋu Matha* (local Indigenous language) as well as in English; to be interactive; and to use a culturally appropriate world view, building on existing knowledge to facilitate shared understandings. An unrelated scoping study [21] that examined ways to improve and support health education and language interpreting in Aboriginal communities in East Arnhem Land (NT) concluded that user-friendly, interactive, tactile, and aesthetically appropriate resources were most successful at facilitating communication. Involvement of the community in the development of resources in a “bottom-up fashion” was also highlighted as crucial to their eventual success. Multimedia resources were felt to be the most useful with a “touch pad body electronic device” being proposed as a tool to facilitate communication around health issues [24].

In Australia, 64.6% of the population owns a mobile phone [25] with the ability to download electronic apps. There has been an explosion in the development and use of apps with 46 billion downloads worldwide in 2012. This figure is estimated to exceed 200 billion/year by 2017 [26], at which time it is predicted that 50% of mobile phone users will have downloaded a health-related app. The potential to harness this technology as a means to improve health literacy, communication, and treatment uptake is yet to be fully realized. Only recently have published articles started to emerge with respect to the evidence base used to develop health-related apps and any subsequent evaluation of their utility and impact [27,28]. Further, only limited literature exists in this field and it raises concerns regarding the accuracy of information [29,30], alignment of advice with evidence-based guidelines [31], and lack of input from users/patients into product design and evaluation of effectiveness [32]. The data with regard to apps specifically targeted at Indigenous or culturally and linguistically diverse groups are even sparser. We are aware of the development of a number of mental health apps and a rheumatic heart disease app (Menzies School of Health Research, Darwin, Australia) specifically for Indigenous Australians but not of any published literature with respect to the development process of health apps for Indigenous populations.

### Participatory Action Research Methodology

Participatory action research (PAR), a cyclical process of reflection, evaluation, and action, where respect for and involvement of the community in all aspects of the research process is an integral part of the methodology, is increasingly recognized as valuable in Indigenous health research [33-35]. There are a number of studies reporting successful outcomes of PAR projects in the context of developing health resources in Indigenous communities [36,37]. This paper aims to describe the process of the development and report the results of the initial evaluation of a culturally appropriate bilingual app about hepatitis B as part of a PAR project.

## Methods

### Overview

This project was undertaken in northern Australia between September 2012 and October 2014. It was based at the health clinic of a remote community in Arnhem Land, 521 km northeast of Darwin (the capital city of Australia’s NT). This community has a population of 2124 with an average age of 24 years, of which 88.98% (1890) are Indigenous Australians and only 9.5% (202) of the population speak English as their first language. On average, there are 4.2 people for each available bedroom and 83.19% (1767) of people live in households considered to be overcrowded. The community has 3 general stores, a school, a library, a police station, and a community church.

Herein we report the results of the second phase of this PAR project. Phase 1 (previously reported in detail [23]) was a qualitative study consisting of semistructured interviews carried out with 3 groups of people, namely, key informants (health clinic staff, community health educators, and liver clinic staff from both urban and remote areas, including doctors and nurses from both Indigenous and nonindigenous groups), Indigenous people living with CHB, and Indigenous community members. Interviews explored the following: background of the individuals, their hepatitis B knowledge, their experience of health communication/education about hepatitis B, available resources, and their perspectives about potentially useful educational tools. The results of phase 1 of the study then formed the evidence base for the development of the bilingual app (phase 2). The original impetus for the project came from the staff of the community clinic. Their enthusiasm for the project led to the development of a collaborative research partnership between the Miwatj Health Aboriginal Corporation Community Clinic (an Aboriginal-controlled health service representing communities across East Arnhem Land), the Royal Darwin Hospital Liver Clinic, and the Menzies School of Health Research.

**Figure 1:**
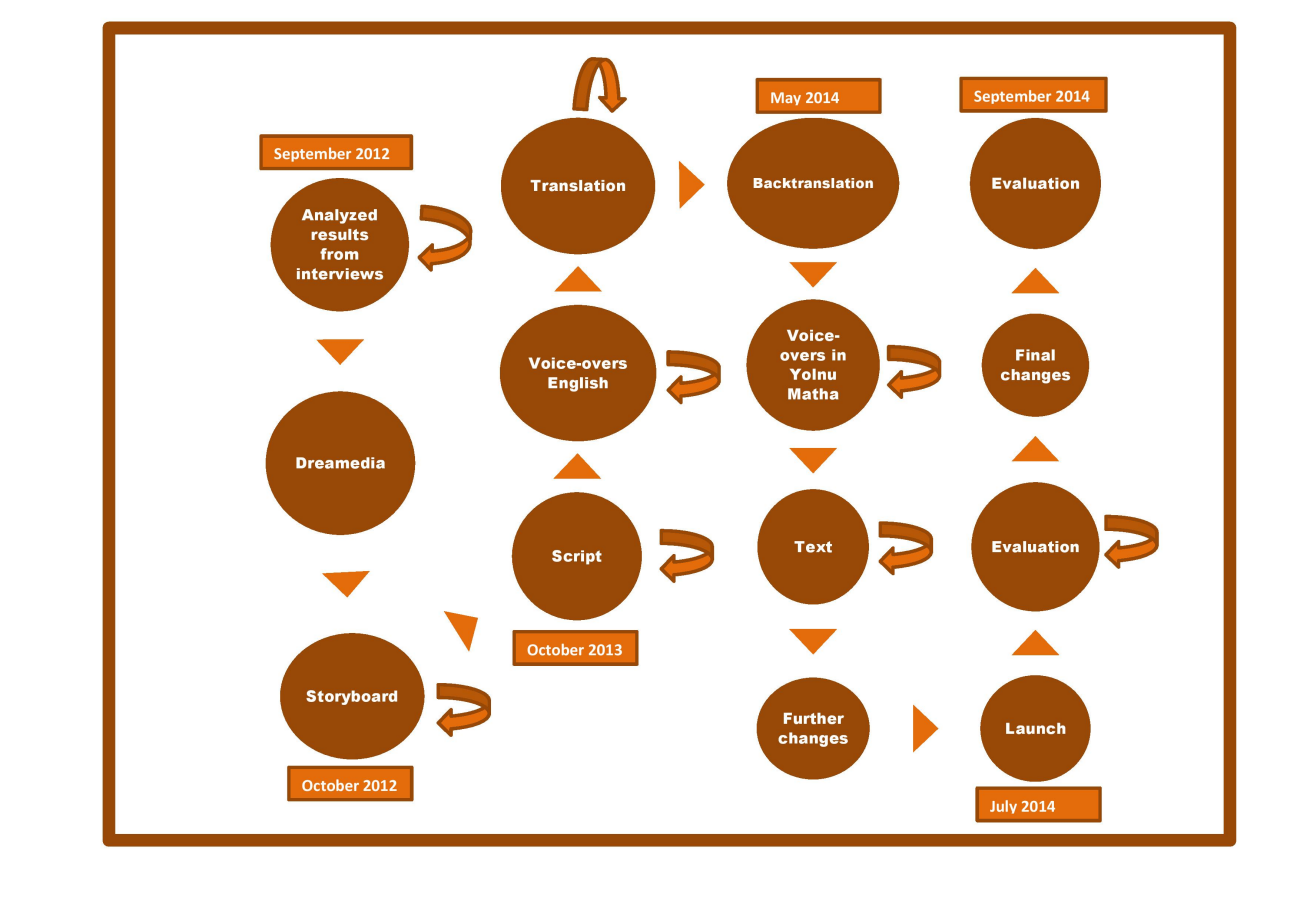
Major stages and timelines for the development of the Hep B Story electronic app. Curved arrows represent time points where episodes of community consultation occurred.

Ethical approval for the study was obtained from the Human Research Ethics Committee of the NT Department of Health and Menzies School of Health Research as well as Miwatj Health Aboriginal Corporation. Figure 1 details the timelines for the development process.

### Development of the Storyboard

**Figure 2:**
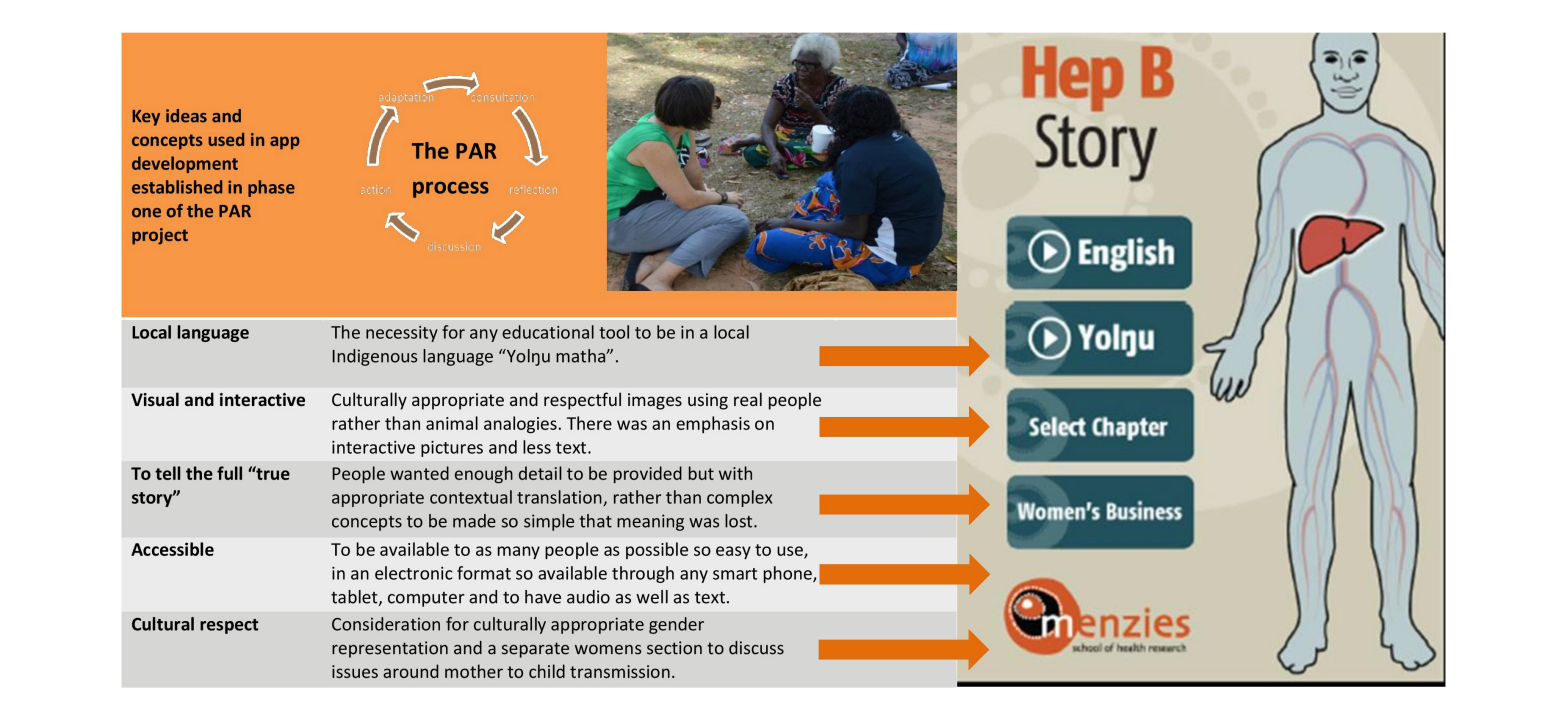
Main concepts and ideas taken from phase 1 [23] of the participatory action research (PAR) process to provide the initial evidence base for the culturally appropriate hepatitis B electronic app. The PAR cycle shows the principle stages involved in a PAR project.

**Figure 3:**
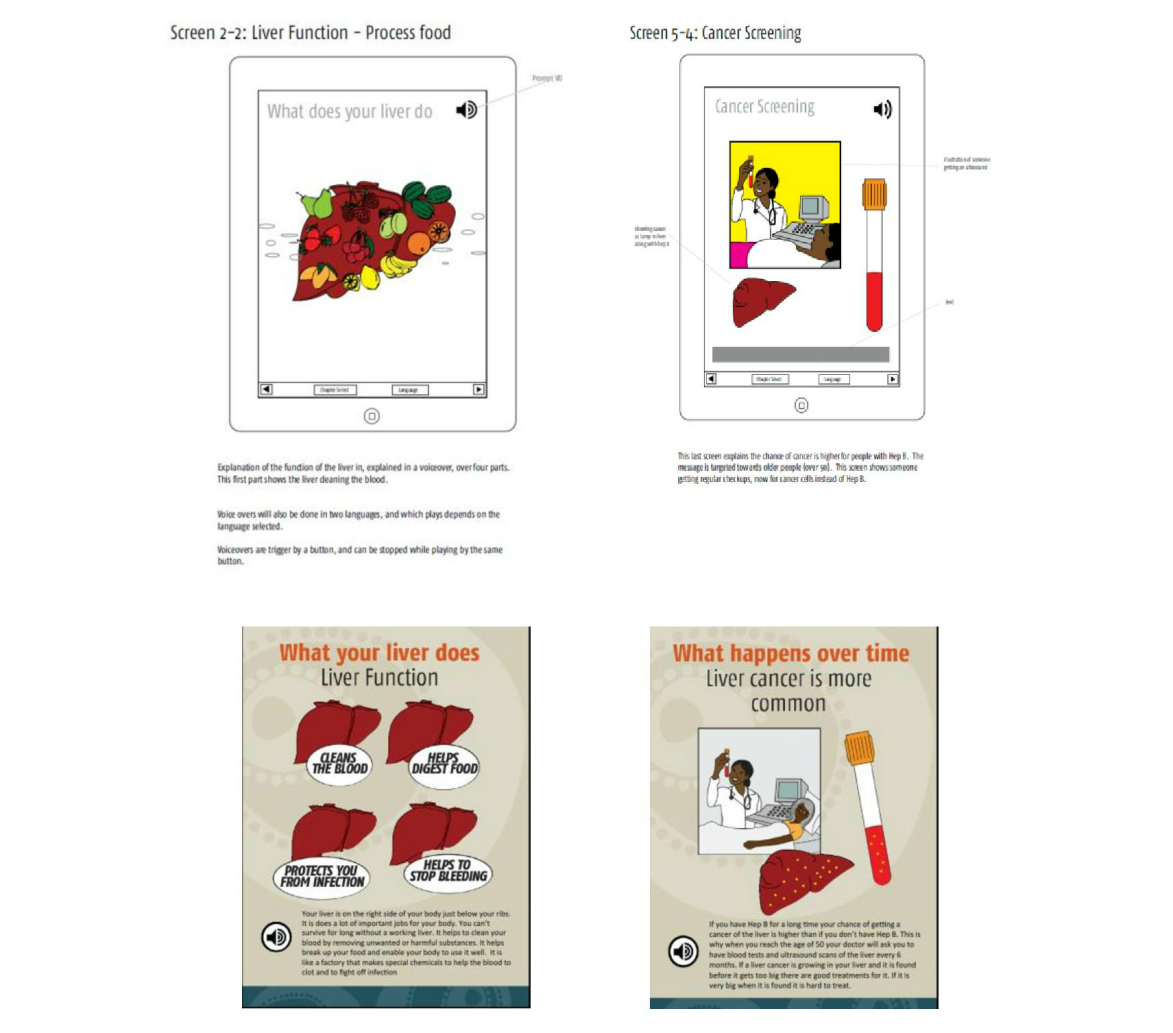
Screen-by-screen examples of the development process showing first and last versions.

Using the specifications and concepts derived from phase 1 of the PAR process (Figure 2), the project team, which included JD, SB, JSD, LC, SS, and VJ, developed an initial storyboard detailing important ideas, images, and themes to be included in the education tool (Figure 3).

This was then developed by Dreamedia, a Darwin-based graphic design and software company, into an initial screen-by-screen storyboard. Subsequently detailed screen-by-screen scripts were developed by the project team, initially in simple English. These preliminary versions of the app were presented back to the community to facilitate and enable evaluation by clinic staff and liver clinic patients. Appropriate modifications were made according to community input. There were in excess of 20 iterations of the storyboard over the period from February 2013 to July 2014.

### Translation Process

The specific language that the app is translated into is “Djambarrpuyngu,” a member of the “Yolŋu Matha” group of Australian Indigenous languages used by Yolŋu people in Northeast Arnhem Land. The term “Yolŋu Matha” will be used in this paper to refer to the language from this point forward as this is the term used in the app in line with community wishes. Two experienced interpreters from the local community were identified, one allocated to do the forward translation (ie, English to Yolŋu Matha) and one to undertake the backtranslation (ie, Yolŋu Matha back to English, so as to check the accuracy of the translation) according to the World Health Organization guidelines [38]. Both interpreters had previously translated health education materials and participated in a pretranslation hepatitis B education session to enable familiarization with the material. Conceptual equivalence (aiming for shared understanding of a word or phrase rather than a word-for-word literal translation) was discussed and encouraged wherever appropriate. Backtranslation was undertaken independently by the second of the interpreters, then the English checked, and clarified by JD (English-speaking doctor with experience in a cross-cultural environment) and SB (bilingual Aboriginal Health Worker), again with emphasis on conceptual and cultural equivalence rather than linguistic equivalence. The final Yolŋu Matha translation was reviewed again by SB. Voice-overs were recorded at Dreamedia studios in both English and Yolŋu Matha.

### Prelaunch Community Review

Functional prototype versions of the electronic app were produced on 4 occasions by Dreamedia and presented to the community for review. Discussion and input from clinic staff (both Indigenous and nonindigenous) and Indigenous liver clinic patients were sought on each occasion. Changes were then incorporated into the next version of the app and the process was repeated until unanimous approval was achieved.

### Launch and Initial Community Evaluation

In July 2014, a launch event was organized in the community, which involved presentation of the app by JD and SB, and an invitation to take part in the initial evaluation process. The evaluation questionnaires had sections to be completed before and after exploring the app. People were guided through the questionnaire process in real time by a bilingual research assistant who translated the questions and answers into Yolŋu Matha where needed or requested.

### Viral Hepatitis Conference Launch and Initial Evaluation

The inaugural Indigenous Peoples’ Conference on Viral Hepatitis in Alice Springs, NT (September 2014) was chosen as an appropriate place to launch the app to the wider sector. The app was presented as part of an exhibit where individuals could explore the app in their own time. We invited all conference delegates to help evaluate the app using the aforementioned questionnaire.

### Evaluation Questionnaire Analysis

Data were entered into Microsoft Excel 2010 (Microsoft, Redmond, WA, USA) and analyzed using Stata version 13 (StataCorp, College Station, TX, USA). Overall preapp and postapp knowledge scores were created by calculating a total score out of 6 (based on the number of correct responses to Q1 and Q4) and presented as percentages. Quantitative continuous variables were presented as mean ± standard deviation for normally distributed parameters and median ± interquartile range (IQR) for non-normally distributed parameters. Bivariate analyses were performed using Student *t* test, paired Student *t* test for preapp and postapp knowledge comparisons, Fisher exact test for comparisons with any cell value less than 5, and Mann-Whitney test for nonparametric data.

Likert-type items (Q2 and Q3) were treated as ordinal data, presented as median values with frequencies, and associations between groups were calculated using Kendall Tau-b [39]. For each Likert type, item 1 equates to “strongly agree, 2 “agree,” 3 “neutral,” 4 “disagree,” and 5 “strongly disagree.” For positive statements, allocated scores were the inverse of the assigned number to enable higher scores to reflect more positive responses.

## Results

### Main Findings

**Figure 4:**
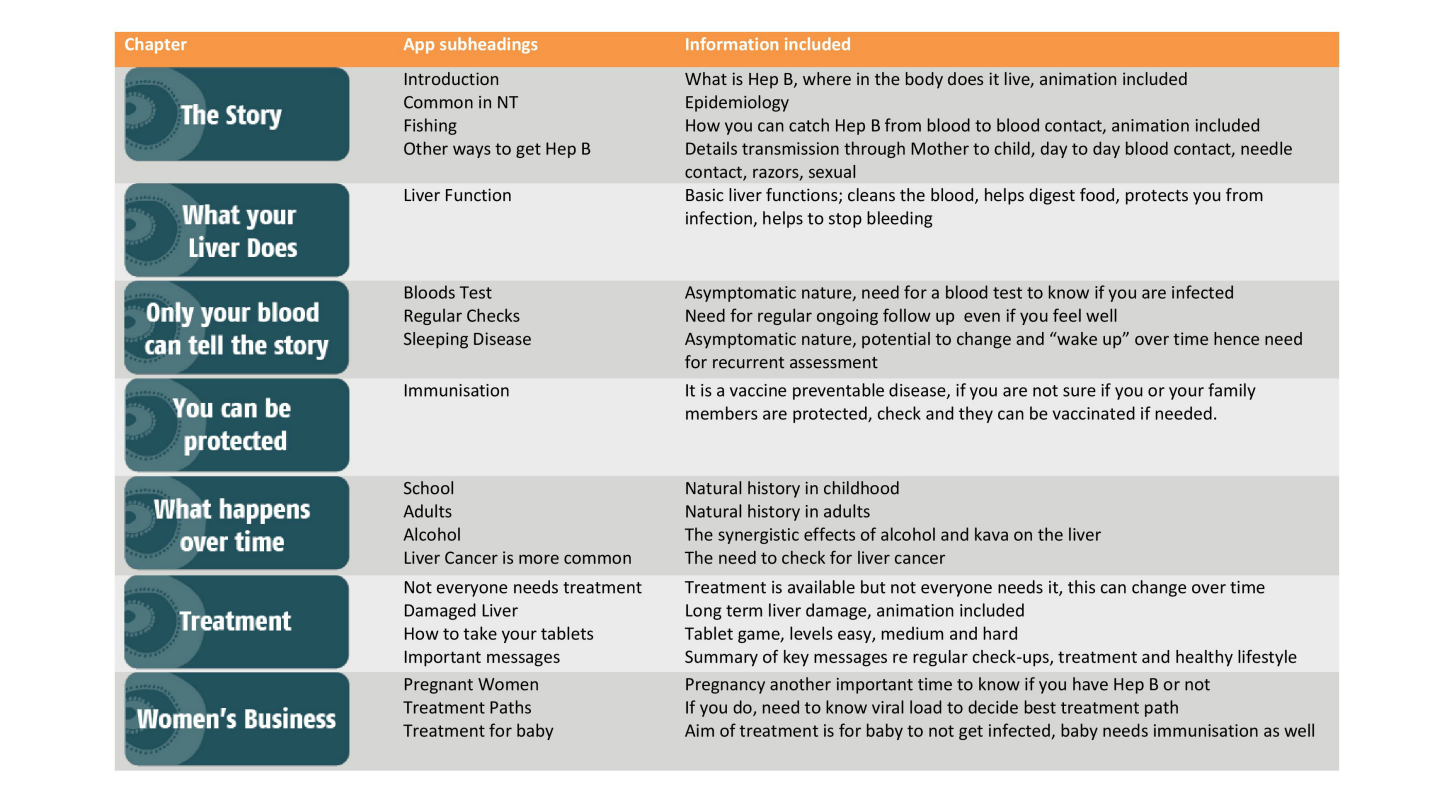
The contents of the Hep B Story app.

The app we produced is called “Hep B Story” and is available as a free download for Apple devices through the Apple App Store, for Android devices through the Google Play Store, and as a Web-based app through the Menzies School of Health Research website. The app’s title screen allows the user to choose the language as either English or Yolŋu Matha and they can navigate from the beginning to the end through the entire app (except for the “women’s business” section), or choose to go to the “chapter select” screen to skip to a specific section or to enter the women’s business section. There are 7 “chapters” as detailed in Figure 4, some of which contain animations. The treatment section includes a game, which involves dragging tablets into a man’s mouth each day. If you do not get him to take the medicine consistently enough, his hepatitis B virus becomes resistant to the tablets and his liver becomes diseased. Each screen has an audio button, which will play the voice-over in English or Yolŋu Matha and the text is also displayed on the screen in the preferred language.

### Development of the Storyboard

As with the text, the images for each screen were discussed in detail over several iterations. Particular emphasis was placed on getting an appropriate balance of gender representation throughout the app, as well as a desire for the individual on the front screen to be gender neutral. Initially, the title screen figure had brown skin, but after community review it was felt that this may imply that all people with hepatitis B have brown skin, and therefore, the color was changed to blue to be ethnically neutral.

The cultural appropriateness of an image to represent sexual transmission was the subject of much discussion. This mode of transmission was, however, downplayed at the community’s request for reasons of stigma and also as the main route of transmission in this population is thought to be mother-to-child transmission during parturition.

The screen representing liver function initially had visual representations of various liver functions (eg, fruit to represent its role in helping to process food). On consultation with the community, this was felt to be a culturally inappropriate use of lateral thinking and was described as a decoy and confusing. People preferred to remove all the pictures and just have the words over the liver images as in the final version (Figure 3).

A separate women’s business section was a recurrent request. It was important to community members that this was not something that you would “stumble upon” while looking through the app and it had to be separate, warranting an active decision to visit this section. “Women’s business” is the commonly used local term for health and other matters specific to women, such as pregnancy.

### Translation Process

The script was translated taking into account context and cultural appropriateness. The Yolŋu Matha version was much longer than the English version (10 pages of text as opposed to 5). There were a number of reasons for this: Yolŋu Matha is a more verbose storytelling language; there is not a direct translation for many terms, for example, “Hep B” in the backtranslation was “those invisible germs that are the sickness in your blood called Hep B.” For clarity, this phrase was then used on each occasion Hep B was mentioned, and therefore increased the number of words. The text was translated meticulously by a senior male Yolŋu elderly person with great attention to detail, contextualization of the text, and cultural appropriateness. For example, the sentence talking about how one of the liver’s functions is to produce clotting factors in the translated version says “it is like a factory that makes good oil, it will help the blood to clot and fight sickness.” A senior female elderly person backtranslated this again with the same attention to detail. Issues raised following the backtranslation were literal interpretation of concepts, the difficulty when there are many words for one thing, and the importance of choosing the most appropriate one for the specific context needed.

### Prelaunch Community Review

One of the key requests from phase 1 of this study was for a visual tool; however, an app prototype without the full text appearing alongside the audio was not well received. The community consensus was to have the full text there so that people could either listen or read depending on their preference.

A request was made to add *kava* drinking (a drink made from the extract of the root *Piper methysticum* with sedative and anesthetic properties, which is commonly used as a recreational drug in parts of Arnhem Land) to the screen about the dangers of the combination of alcohol and hepatitis B for the liver. An image of a family with several members infected was also suggested to be included on the screen talking about epidemiology.

### Launch and Initial Community Evaluation

The launch in the community was met with excitement and pride at seeing an electronic app in Yolŋu Matha. The evaluation questionnaire was completed by 16 people, median age 34 years (IQR 30-59), with 12 (75%) being women. Results are presented in Tables 1 and 2.

### Viral Hepatitis Conference Launch and Initial Evaluation

The launch and 3-day exhibit at the Viral Hepatitis Conference in Alice Springs, NT (September 2014) were also met with a positive response. The questionnaire was completed by 56 people, median age 45 years (IQR 36-54), with 50 (89%) being women. Results are presented in Tables 1 and 2.

**Table 1:** Demographics of participants and results of the opinion-based component of the evaluation questionnaire.

		Community launch group (N=16)	Conference delegate group (N=56)	*P* value^a^
**Demographics of groups**				
	Age in years median (interquartile range)	34 (30-59)	45 (36-54)	.34
	Indigenous status (%)	94 (15/16)	21 (12/56)	<.001
	Female gender (%)	75 (12/16)	89 (50/56)	.20
**Self-rated hepatitis B knowledge (% who strongly agree or agree)** ^a^				
	Never heard of hepatitis B	50 (8/16)	4 (2/56)	<.001
	I knew a lot about hepatitis B	31 (5/16)	66 (37/56)	.02
**Postapp opinion using the 5-point Likert scale Median score, % giving that answer (n/N)** ^b^				
	I found the app easy to use (for Q3a)	5, 67 (10/15)	5, 80 (44/55)	.29
	Easy to understand (for Q3b)	5, 67 (10/15)	5, 75 (41/55)	.43
	Contained enough information for my needs (for Q3c)	5, 47 (7/15)	5, 67(37/55)	.42
	Contained too much information for my needs (for Q3d)	2, 50 (6/12)	4, 17 (9/54)	.005
	I would recommend the app to my family and friends (for Q3f)	5, 69 (9/13)	5, 67 (36/54)	.72
	Use the app again myself (for Q3g)	5, 64 (9/14)	5, 55 (29/53)	.56

^a^ *P* values are comparisons between the community launch group and the conference delegate group.

^b^“Self-rated hepatitis B knowledge” and “Postapp opinion” constituted the results of opinion-based questions.

**Table 2:** Results of the knowledge-based component of the evaluation questionnaire.

Knowledge-based questions	Community launch group (n=16)^a^			Conference delegate group (n=56)^a^		
	Preapp	Postapp	*P* value^b^	Preapp	Postapp	*P* value^b^
Can you name 3 ways by which hepatitis B can be passed from one person to another?	33 (16-50)	58 (39-77)	.01	100 (100-100)	97 (96-100)	.32
If you have hepatitis B what is the best way to tell if the virus is causing damage to your liver?	25 (1-49)	38 (11-64)	.16	92 (85-99)	89 (83-99)	.56
If you have hepatitis B what can you do to help your liver to stay healthy (name 2 things)?	47 (29-65)	50 (28-72)	.58	84 (76-93)	94 (88-99)	.02

^a^Values are presented as mean score % (95% CI).

^b^ *P* value for paired *t* test comparing preapp versus postapp knowledge scores.

### Evaluation Questionnaire Results

Overall, 72 individuals completed the evaluation questionnaire of whom 62 (86%) were women and 27 (38%) were Indigenous Australians, with a median age of 44 years (IQR 34-54). There was a good representation of individuals from most parts of Australia with only Tasmania and Australian Capital Territory not being represented. With regard to preapp and postapp knowledge assessment, there was a statistically significant increase in the first knowledge-based question in the community launch group (*P*=.01) and in the final one in the conference delegate group (*P*=.02, Table 2).

Participants’ opinions of the app after use were generally positive, with 5 of the 6 questions on this achieving a median rating of 5 on the 5-point Likert-type scale. The free text comments were overwhelmingly positive with multiple references to ease of use, culturally appropriate graphics, and the importance of being able to both read and listen in Yolŋu Matha. Multiple requests were made for the app to be translated into other languages. A recurrent criticism was made referencing the lack of inclusivity with regard to gender and sexually diverse communities by the wider key informant group.

## Discussion

### Principal Findings

We describe in detail the process of development of the “Hep B Story” app, a culturally appropriate educational resource about CHB, through a community partnership using a PAR framework. “Hep B Story” is the first app to be produced in Djambarrpuyngu, a member of the Yolŋu Matha group of Australian Indigenous languages spoken widely in the North East Arnhem Land in Australia’s NT. The continuous iterative cycle of consultation, evaluation, and adaptation has led to the production of a tool that the community was proud of and excited about. Initial evaluations from both the community and a wider group of key stakeholders have been overwhelmingly positive.

The production of apps and literature concerning their development are rapidly increasing; however, for the vast majority of health-related apps, there is currently no standardized development or regulatory process for assessment of their quality or effectiveness [27]. There are few published descriptions of the process of health app development and it is often difficult to ascertain, when considering using or recommending an app, who exactly has produced the content and if end users have been involved in the process. Recent reviews of apps for specific disease areas have highlighted concerns regarding factual accuracy, lack of end user involvement, and the effectiveness of apps to add value to standard health care practice [28]. A Cochrane review [31] looking specifically at apps facilitating the self-management of asthma (>100 apps available) concluded that the current evidence base (only 2 studies included) is not sufficient to advise clinical practitioners, policy makers, or the general public regarding app effectiveness. A number of reviews on dermatology apps directed toward skin cancer screening have reported wide ranges of sensitivity and specificity for the diagnosis of melanoma [30,40], with one reporting that 88.2% of biopsy-proven melanoma was classified by the app as “medium risk” and individuals were thus advised to “monitor only” [41]. A trial protocol has been published for an evaluation of the effectiveness of a suicide-prevention app in Indigenous Australian youth (currently recruiting); however, no details are currently available as to how the app was developed [42]. Recently, regulations have been introduced by both the United States Food and Drug Administration and the European Union for “medical device” apps (those intended as an accessory to a regulated medical device or those that transform a mobile platform into a regulated medical device, such as an app intended to diagnose cardiac arrhythmias). These regulations do not currently apply to health information or education apps [43]. A number of app clearinghouse websites are now available with varying levels of review and accreditation of health-related apps [27].

In the context of Indigenous health, community partnerships and the use of PAR methodologies have been shown to help break down barriers to communication [34,36], and understandably people respond to information in their own language more positively than that in a second language. Our experience highlights the importance of meticulous translation, backtranslation, and the attention to detail needed to ensure that the messages you are giving are both linguistically and contextually correct and will be understood in the way intended. There is great potential for harm if this process is not robust. This was highlighted in phase 1 of the PAR process when during the process of the interviews it became apparent that there was a lack of shared understanding of the word “silent” between nonindigenous health workers and Indigenous patients in the context of hepatitis B. The health workers used this to describe the asymptomatic nature of hepatitis B noted at times, whereas the patients understood this to mean that the sickness is brought about by sorcery [23].

We also highlight the great value that can be gained from repeated reviews of and conversations about a product throughout the development process, particularly having a real prototype version of the app to comment on. This allowed multiple changes and additions to the visual appearance and aesthetics of the app such as the color of the person and the images on the liver function screen, which would have been difficult to tease out from verbal-only communication. It also provides multiple opportunities to open up communication facilitating collective agreement making and allowing “bottom-up” changes to occur, which have been highlighted as crucial to change in the context of remote Indigenous communities [24].

Initial evaluations of the Hep B Story app were overwhelmingly positive, with all but 1 question (question 3d) assessing user’s opinions achieving a median rating of 5 on a 5-point Likert-type scale. Question 3d was a negative statement compared with all the others, which were positive and it may be that this was confusing or difficult to translate as the lowest score for this question came from the community evaluation. This needs further clarification and exploration before the design of any subsequent evaluation. It will also be important to consider why the numbers of people completing the questionnaire in the community launch group were much lower than the conference delegate group. It may be that due to different cultural and communication protocols, as a questionnaire-based evaluation is not the optimal methodology to use in this setting.

There was a significant increase in knowledge after use of the app for questions 1a in the community launch group (*P*=.01) and 1c in the conference delegate group (*P*=.02). Although the mean score increased from 25% to 38% in the community launch group for knowledge question 1b, this was not statistically significant (*P*=.16), and it is possible that this would reach significance with a larger sample size. It is also important to acknowledge the persistence of low levels of knowledge in the community launch group even after exploring the app and pertinent to critically examine whether this is likely to be a problem with the app itself or the way the questions were asked and the methodology used in this initial evaluation. Culturally appropriate measurement of the impact of interventions aimed at improving health knowledge is problematic, with a tendency to attempt to quantify “knowledge” without having any validated tools to achieve this in an Indigenous setting. There are a number of studies in progress in an Australian Indigenous setting using adapted versions of validated health literacy measurement tools such as the Health Literacy Management Scale and Health Literacy Questionnaire, which will hopefully provide much needed information in this area [44-46]. Christie and Verran [24] have suggested that, in the context of East Arnhem Land, low health literacy is not so much a knowledge problem but a need for allowing shared understandings to develop, and our work from the first phase of this PAR project would generally concur with this [23]. It is, therefore, worthwhile considering the cultural appropriateness of any kind of “knowledge measurement” within our further evaluation. It is important that a larger more formal evaluation of this app does take place to confirm and add more detail regarding the impact on knowledge and ultimately behavior change; however, it may be that an interview-based evaluation methodology is more appropriate. Because of the nature of the PAR process, it would be difficult to objectively evaluate this app’s effectiveness and acceptability in a community that has already been so involved in its development. We are therefore planning a separate evaluation in a different location where Yolŋu Matha is spoken and hepatitis B is common.

Even in a community where there has been significant engagement, involvement, and interest in the hepatitis B project, levels of knowledge around CHB were low with 50% (8/16) of people having never heard of it. This is consistent with the work done in the Torres Strait region of Australia [19,22], which reported a lack of awareness and/or knowledge of CHB and the measures to reduce its health impact both at the patient and at the health care provider level. The free text comments from the conference delegate group with respect to gender and sexually diverse communities highlighted the conflicts that can arise in PAR projects. Obtaining the right balance between cultural appropriateness and community wishes versus inclusivity of potentially underrepresented groups in this context can be challenging.

Limitations to extensive engagement and involvement of end users in the process of app development include the length of time needed to undertake this process robustly, particularly in communities where English is not the first language, and worldviews of health are very different. This is especially relevant to digital technologies where the pace of change is so fast. As an intended consequence of the methods used to produce the app, it is very specifically tailored to the culture and needs of Yolŋu people; therefore, its translatability to other groups is always going to require further consultation and adaptation. Although this process can be streamlined when the framework and technical coding for the app are available, it is still a significant undertaking. Another obvious issue is that not everyone has access to a mobile phone or tablet device. As such, we felt it crucial to also have the app available through an Internet site that can be accessed from nonportable computers such as those in health care facilities.

The potential for adaptation to other languages is important in the context of hepatitis B, which disproportionately affects Indigenous people both in Australia and across the world. There is also great potential to personalize and incorporate clinical tools into the app such as tracking of blood test results, triggers for follow-up, medication reminders, and even self-assessment tools. One example would be adapting the “number connection test,” which is a widely used method for detecting the reduced spatial awareness and coordination present in hepatic encephalopathy. This currently consists of a timed test connecting numbered dots on a sheet of paper, which could be adapted into a game-style version and included in the app. The section about alcohol and kava could incorporate harm-reduction strategies, monitoring of consumption, and abstinence encouragement tools.

### Conclusions

Health-related apps have a huge potential to contribute and impact positively on health care; however, there is also substantial risk of harm in the absence of an evidence base to guide standards, regulation, and development. This is particularly true for populations where health beliefs and worldview are different and English is not the first language. These are the very populations that are most affected by CHB. We described using a PAR framework with both end users and key informants providing their inputs into the content and development of a hepatitis B-specific app. Although this process was time consuming, the approach was very successful, with a majority of participants providing positive responses. The long-term effectiveness with respect to improving patient’s health literacy leading to behavior change and increased treatment uptake will be evaluated over time.

## Abbreviations

CHB: chronic hepatitis B
IQR: interquartile range
NT: Northern Territory
PAR: participatory action research
